# Evolutionary history of the genus *Tarentola *(Gekkota: Phyllodactylidae) from the Mediterranean Basin, estimated using multilocus sequence data

**DOI:** 10.1186/1471-2148-12-14

**Published:** 2012-01-30

**Authors:** Catarina Rato, Salvador Carranza, David J Harris

**Affiliations:** 1 CIBIO, Centro de Investigação em Biodiversidade e Recursos Genéticos, Campus Agrário de Vairão, 4485-661 Vairão, Portugal; 2 Departamento de Biologia, Faculdade de Ciências da Universidade do Porto, 4099-002 Porto, Portugal; 3 Institute of Evolutionary Biology (CSIC-UPF)-Passeig Marítim de la Barceloneta, 37-49, E-08003 Barcelona, Spain

## Abstract

**Background:**

The pronounced morphological conservatism within *Tarentola *geckos contrasted with a high genetic variation in North Africa, has led to the hypothesis that this group could represent a cryptic species complex, a challenging system to study especially when trying to define distinct evolutionary entities and address biogeographic hypotheses. In the present work we have re-examined the phylogenetic and phylogeographic relationships between and within all Mediterranean species of *Tarentola*, placing the genealogies obtained into a temporal framework. In order to do this, we have investigated the sequence variation of two mitochondrial (12S rRNA and 16S rRNA), and four nuclear markers (ACM4, PDC, MC1R, and RAG2) for 384 individuals of all known Mediterranean *Tarentola *species, so that their evolutionary history could be assessed.

**Results:**

Of all three generated genealogies (combined mtDNA, combined nDNA, and mtDNA+nDNA) we prefer the phylogenetic relationships obtained when all genetic markers are combined. A total of 133 individuals, and 2,901 bp of sequence length, were used in this analysis. The phylogeny obtained for *Tarentola *presents deep branches, with *T. annularis, T. ephippiata *and *T. chazaliae *occupying a basal position and splitting from the remaining species around 15.38 Mya. *Tarentola boehmei *is sister to all other Mediterranean species, from which it split around 11.38 Mya. There are also two other major groups: 1) the *T. mauritanica *complex present in North Africa and Europe; and 2) the clade formed by the *T. fascicularis*/*deserti *complex, *T. neglecta *and *T. mindiae*, occurring only in North Africa. The cladogenesis between these two groups occurred around 8.69 Mya, coincident with the late Miocene. Contrary to what was initially proposed, *T. neglecta *and *T. mindiae *are sister taxa to both *T. fascicularis *and *T. deserti*.

**Conclusions:**

At least in the Iberian Peninsula and Northwest Africa, the lineages obtained have some geographic coherency, whilst the evolutionary history of the forms from Northeast Africa remains unclear, with a paraphyletic *T. fascicularis *with respect to *T. deserti*. The separation between the *T. mauritanica *complex and the clade formed by the *T. fascicularis*/*deserti *complex, *T. neglecta *and *T. mindiae *is coincident with the uplift of the Atlas Mountain chain, and the establishment of two distinct bioclimatic regions on each side of the barrier.

## Background

The Mediterranean Basin has been the stage of various paleogeographical events that have helped shape the genetic diversity and phylogeographic patterns of numerous taxa. In particular, the connection between the Mediterranean Sea and the Atlantic Ocean closed about 5.96 million years ago (Mya) causing the Mediterranean Sea to desiccate during the Messinian Salinity Crisis, an event that ended 5.33 Mya, with the formation of the Strait of Gibraltar [1-3]. The opening of the Strait led to the separation of terrestrial faunal elements in the region into allopatric units [3], as in the cases of the spiny-footed lizards *Acanthodactylus erythrurus *[4], or the West Mediterranean newts of the genus *Pleurodeles *[5]. Regarding the study performed on the spiny-footed lizards, the Iberian *Acanthodactylus e. erythrurus *apparently became separated from its North African relatives around 5 Mya [4], and it was also the refilling of the Strait of Gibraltar that may have led to the separation between *Pleurodeles waltl *and the North African newts *Pleurodeles nebulosus *and *Pleurodeles poireti *[5]. Another extremely influential event occurred during the Pleistocene when sea level and climate oscillations led to repeated isolation and connection of taxa [6,7], and consequent population differentiation [e.g. [8,9]]. At a more local scale, the Atlas Mountains originated during the mid- to late Miocene as a consequence of the impact between the Eurasian and African plates [10], and seem to have been an important barrier for allopatric speciation [e.g. [11,12]]. All these events have made the Mediterranean Basin a centre for genetic differentiation that, in some cases, has led to vicariant speciation, partially explaining the observed high levels of biodiversity [13,14].

*Tarentola *geckos are members of the Family Phyllodactylidae [15], and the genus is currently comprised of 21 different species [16-20]. *Tarentola *is distributed across the Mediterranean Basin and on many Macaronesian islands, including Madeira, the Selvages, the Canary and Cape Verde islands [21,22]. On the other side of the Atlantic Ocean, three species are accepted: *T. americana*, from Cuba and the Bahamas; the recently described *T. crombiei *[18] endemic to Cuba; and the probably extinct *T. albertschwartzi *[16], known from a single specimen allegedly from Jamaica. Since all the Macaronesian islands are volcanic, and therefore have never been connected to the mainland [23], the most likely hypothesis is that these geckos reached these islands by transmarine dispersal [21,22]. Regarding the Neotropical members of *Tarentola*, these also seem to be the result of a post-Gondwanan dispersal from the Old World [21,24,25].

The Mediterranean Basin harbours nine different species of *Tarentola*, namely *T. mauritanica, T. chazaliae, T. deserti, T. boehmei, T. annularis, T. neglecta, T. mindiae, T. ephippiata*, and *T. fascicularis*, a former subspecies of *T. mauritanica *recently elevated to the species level [17]. Although several molecular studies have been published for some of these species, these have always been focused on one [26-30] or a few taxa [17,19,21,24,25]. Moreover, all species of Mediterranean *Tarentola *have never been assembled into a phylogenetic context using a multilocus approach. The majority of the studies have been focused on the phylogeographic patterns of the Moorish gecko *T. mauritanica*. This species is characterized by an extremely high mitochondrial genetic variation in North Africa, which led to the hypothesis that this taxon could be, in fact, a species complex [29,30]. In contrast, the European populations of the Moorish gecko have very low mitochondrial diversity, initially thought to be the result of a recent introduction [21,28-30], probably human-mediated. However, a recent study [26] has demonstrated that this low mtDNA variability that characterizes the European populations of *T. mauritanica *could be the result of genetic hitch-hiking, and not solely due to a recent colonization event.

Additionally, several studies have demonstrated the paraphyly of *T. mauritanica *with respect to *T. angustimentalis *from the Canary Islands [21,26-30]. A recent study [17] concluded that populations of *T. fascicularis *from Libya and central Tunisia constitute a monophyletic lineage and, together with *T. mindiae *and *T. neglecta*, represent the sister group of *T. deserti*. On the other hand, previous mtDNA studies indicated that *T. fascicularis *is paraphyletic with respect to *T. deserti *[26-30], although sampling was limited in all cases.

Therefore, in order to estimate an evolutionary hypothesis able to explain the previously mentioned patterns we have increased the taxonomic and geographic sampling in this study. Further, we used two mitochondrial, and four nuclear markers in order to better evaluate the inter- and intraspecific relationships within the genus *Tarentola *from the Mediterranean Basin.

## Results

### mtDNA genealogy

**Figure 1:**
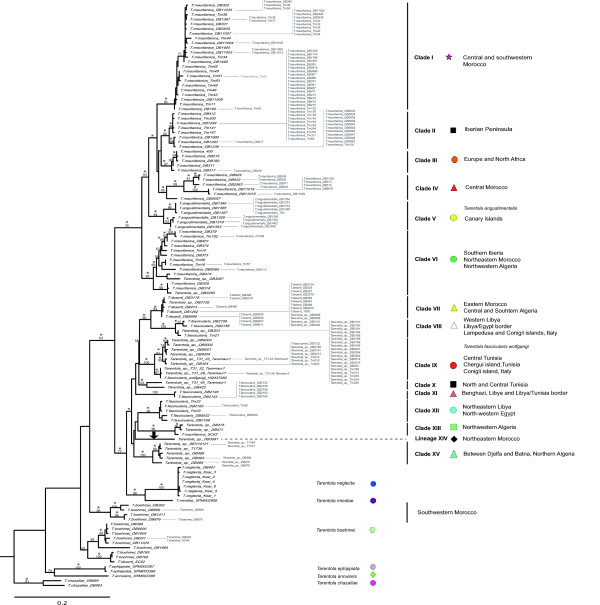
**RAxML mtDNA phylogenetic tree for the combined 12S rRNA and 16S rRNA**. Maximum likelihood bootstrap scores are represented over key nodes, and the star symbol corresponds to Bayesian posterior probabilities higher than 95%. The black arrow corresponds to incongruences between both phylogenetic methods, with DB3091 appearing as sister taxa to Clade XII, with Bayesian Inference, also weakly supported (53%).

**Figure 2:**
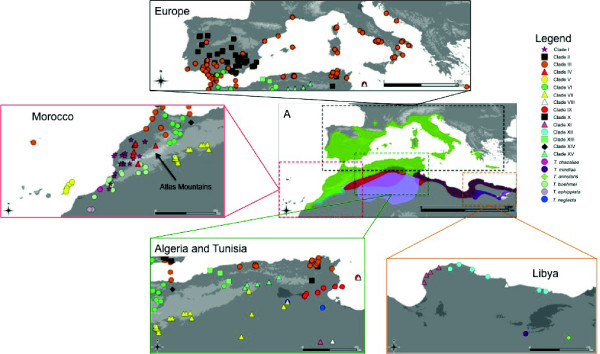
**Maps highlighting distinct regions of *Tarentola*'s geographic distribution, where all sampled specimens used in this study are represented**. Each symbol corresponds to the mtDNA clades. Colours in map A correspond to the geographic distribution of several species of the genus *Tarentola *according to IUCN [13]: the green colour corresponds to *T. mauritanica*; the light blue to *T. boehmei*; the red to *T. deserti*; the light purple to *T. neglecta*; and the dark purple to *T. mindiae*. The brown colour corresponds to the distribution of *T. fascicularis *according to the Reptile DataBase www.reptile-database.org. Regarding *T. chazaliae, T. annularis *and *T. ephippiata *the geographic ranges of all these species are given in [32,33].

With the program ALTER [31] the 384 concatenated mitochondrial sequences were reduced to 125 haplotypes used in the analyses. A total of 818 bp (132 variable sites), corresponding to 301 bp of 12S rRNA and 517 bp of 16S rRNA, were used. According to jModelTest the model of nucleotide substitution that best fits the 12S rRNA dataset is the SYM+I+G, and for the 16S rRNA it is the HKY+G. According to the obtained mitochondrial DNA genealogy results (Figure 1), we were able to recover six major groups within the Mediterranean species of *Tarentola*, geographically represented in Figure 2; one group corresponding to *T. chazaliae*; another one clustering *T. annularis *and *T. ephippiata*; *T. boehmei *appears as two separated lineages; another clade comprised of *T. neglecta *and *T. mindiae*, sister taxa to both *T. deserti *and *T. fascicularis*; and another group clustering *T. mauritanica *and *T. angustimentalis *with this latter rendering the former paraphyletic, as previously reported [21,26-30]. Within these major divisions *T. chazaliae *is basal to all Mediterranean *Tarentola *and *T. annularis *and *T. ephippiata *are sister taxa. Within the *T. boehmei *group considerable diversity was obtained, with one lineage from southwestern Morocco appearing as an independent evolutionary entity, sister to all remaining *Tarentola*, although this relationship was not statistically supported. In fact, the southwestern Moroccan clade of *T. boehmei *is the only one presenting strong geographic coherency, with the remaining lineages of this species clustering specimens from different geographic localities. A further feature of the *T. boehmei *lineage is the existence within this clade of a specimen assigned as *T. deserti *(SC62) by Carranza *et al. *[19]. In fact, SC62 was collected from the same locality as Td55, a specimen morphologically identified as *T. deserti*, and that falls within the *T. deserti *clade [29], and far away from the distribution range of *T. boehmei*. Unfortunately, there is no available information for the nuclear markers of this specimen, in order to determine if this is a case of hybridization, mitochondrial introgression between *T. boehmei *and *T. deserti*, a misidentification (implying a substantial range extension) or some other form of error.

The group comprised by *T. mauritanica *and *T. angustimentalis *can be subdivided into six different evolutionary clades (I to VI); Clade I is present in Central and southwestern Morocco; Clade II corresponds to the exclusive Iberian lineage first identified by Perera and Harris [28]; Clade III is present throughout Europe and North Africa; a new clade assigned as Clade IV occupies Central Morocco; the *T. angustimentalis *(Clade V) lineage within the *T. mauritanica *complex; and finally Clade VI distributed across southern Iberia, northeastern Morocco and northwestern Algeria. Hereafter, this major group is referred to as the "*T. mauritanica *complex".

When analysing the last major lineage, there is evidence that *T. neglecta *and *T. mindiae *are sister taxa to both *T. fascicularis *and *T. deserti*, rather than the previous estimate of relationships [17], in which *T. neglecta*, together with *T. mindiae *and *T. fascicularis*, were sister taxa to *T. deserti*. A paraphyletic *T. fascicularis *with respect to *T. deserti*, as already obtained in previous studies, [26-30] was also recovered. This major group that hereafter is referred to as the "*T. fascicularis*/*deserti *complex" was subdivided into nine different lineages (from VII to XV), characterized by little geographical and clear phylogenetic structure. Specimens from Clade VII occur from eastern Morocco to Central and southern Algeria, fitting the known distribution of *T. deserti *[32,33]. In fact, the specimens from this study collected in the Algerian Sahara (DB481 and DB488) are located in the north and south border of the type locality of this species (Ouargla, Algerian Sahara) [34]. The sister lineage of Clade VII is Clade VIII present in western Libya, Lampedusa and Conigli islets (Italy), and on the border between Libya and Egypt, morphologically assigned to *T. fascicularis *(P. Geniez, pers. comm.). Clade IX ranges from Central Tunisia to Chergui island (Tunisia) and Conigli islet in which the recently described subspecies *T. fascicularis wolfgangi *[17] from central Tunisia clusters with the remaining specimens. Clade X ranges from North to Central Tunisia, and Clade XI is present in the Benghazi region (Libya), and on the Libya/Tunisian border. The type locality of *T. fascicularis *is Ain Teyanah, 20 km South of Benghazi [35], and specimens from Clade XI are included in this area. Clade XII is present in northeastern Libya and northwestern Egypt, while specimens from Clade XIII occur in northwestern Algeria. Lineage XIV is represented by a unique individual (DB3091) from northeastern Morocco, and Clade XV occurs in northern Algeria between Djelfa and Batna.

This same topology was obtained with both phylogenetic methods, except regarding the specimen DB3091 that appears as sister taxon to Clade XII with the Bayesian Inference (BI).

### nDNA genealogy

**Figure 3:**
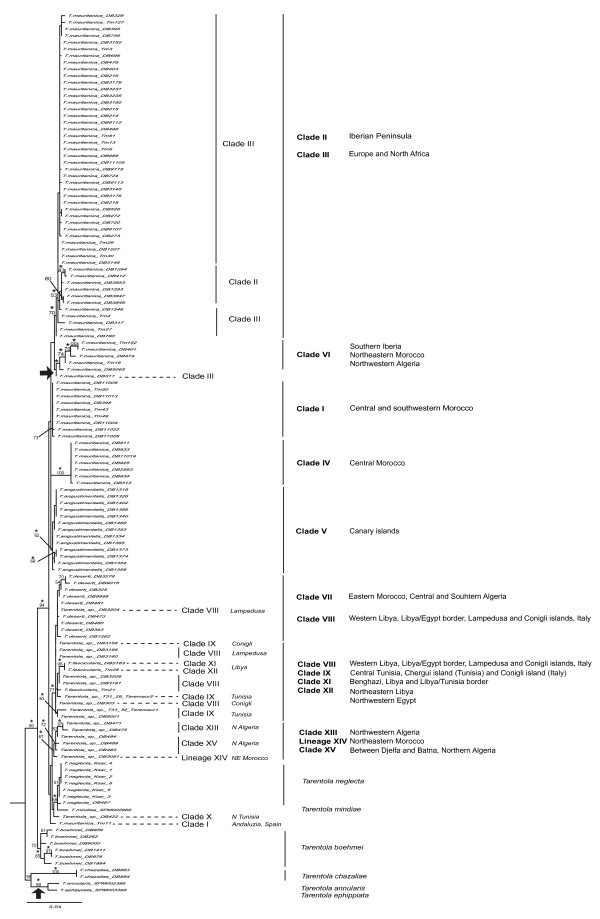
**RAxML phylogenetic tree for all concatenated nuclear markers, namely ACM4, MC1R, PDC, and RAG2**. Maximum likelihood bootstrap scores are represented over key nodes, and the star symbol corresponds to Bayesian posterior probabilities higher than 95%. The black arrows correspond to incongruences between both phylogenetic methods, with DB311 appearing as sister taxon to Clades II and III (with 67% of posterior probability), and *T. annularis *and *T. ephippiata *(without *T. chazaliae*) with a basal position in the phylogeny, according to Bayesian Inference. According to the BI method, *T. chazaliae *is separated from the remaining *Tarentola *with a 64% of posterior probability.

Concerning the phylogenetic inference with solely the four nuclear markers (Figure 3), we used a total of 133 individuals, with a combined length of 2,084 bp; 368 bp for the ACM4, 602 bp for the MC1R, 363 bp for the PDC, and 751 bp for the RAG2. The nucleotide models obtained for each gene were the GTR+G for the ACM4, the GTR+I+G for both MC1R and RAG2, and the K80+G for the PDC. The topologies obtained from both phylogenetic methods (ML and BI) were exactly the same, except regarding the specimen coded as DB311, which appears as sister taxon of Clades II and III in the BI analyses, and the clade clustering *T. annularis *and *T. ephippiata*, which is estimated to be the sister taxa of all other ingroup lineages using BI.

From the analysis of the nuclear dataset, four of the six major lineages identified in the mitochondrial phylogeny are recovered. However, the phylogenetic relationships within each of these lineages are quite different, relative to the ones obtained with the mtDNA dataset, and several of them are poorly supported with multiple polytomies. According to the ML analyses, *T. ephippiata *and *T. annularis *are sister taxa to *T. chazaliae*, although poorly supported. Also, the two mtDNA independent lineages of *T. boehmei *are recovered as monophyletic and contrary to the mtDNA phylogeny is considerably supported (70% bootstrap). The Iberian clade (Clade II) within Clade III renders the latter paraphyletic and, therefore, they could be considered a single lineage based on the nDNA data. Clade VI appears as the sister taxa of both Clades II and III, instead of Clade IV. Relationships between Clades I, IV and V are unresolved. The group comprised by the *T. fascicularis*/*deserti *complex, *T. neglecta *and *T. mindiae *contains a single individual from Clade I (Tm11) that is more related to these taxa, rather than to the specimens from the *T. mauritanica *complex. This major group is characterized by several very poorly supported lineages. Clade VII is recovered, although an individual from Clade VIII (DB3204) makes this lineage paraphyletic. The *T. mindiae *and *T. neglecta *clade is again recovered as a lineage, with an individual from Clade X (DB422) as sister taxon to this lineage. Finally, all North Algerian, and northeastern Moroccan mtDNA lineages (clades XIII, XIV, and XV) were recovered as a distinct lineage by the nDNA.

### Combined analysis (mtDNA+nDNA) and age estimates

**Figure 4:**
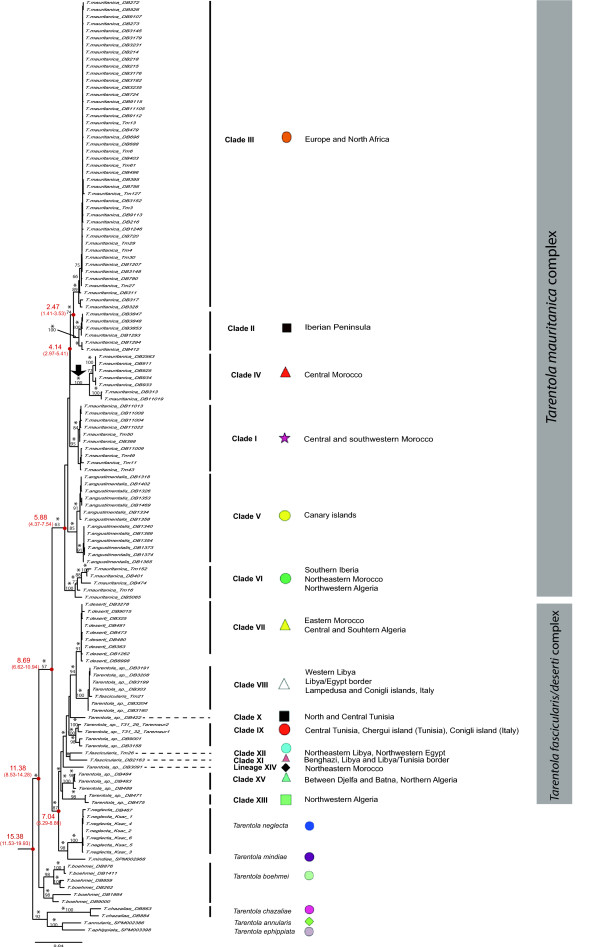
**RAxML phylogenetic tree for all markers used in this study (mtDNA+nDNA)**. Maximum likelihood bootstrap scores are represented over key nodes, and the star symbol corresponds to Bayesian posterior probabilities higher than 95%. Below the nodes, and in red are represented the estimates (mean and 95% credibility interval) obtained of the time to the most recent common ancestor (TMRCA). The black arrow corresponds to incongruences between both phylogenetic methods, with Clade IV appearing as a politomy and sister taxa to Clades I and V, according to the Bayesian Inference.

In order to perform this analysis (Figure 4 and Additional file 1, Figure S1), we used a total of 133 individuals, and 2,901 bp (817 bp of two mtDNA genes, and 2,084 bp of four nDNA genes). All lineages identified with the mtDNA dataset were recovered, although some of the relationships between them were altered. According to this genealogy, *T. annularis *and *T. ephippiata *appear as sister taxa, in turn related to *T. chazaliae*. These three species split from the remaining Mediterranean *Tarentola *approximately 15.38 (11.53-19.93) Mya. The *T. boehmei *lineage appears as monophyletic and originated around 11.38 (8.53-14.28) Mya. Within the *T. mauritanica *complex, Clade I is sister taxon to Clades II, III and IV, and all six lineages from this major group originated between 5.88 (4.37-7.54) and 2.47 Mya (1.41-3.53), corresponding to the late Miocene and late Pliocene, respectively. As obtained from the mtDNA dataset alone, Clade VI is the sister taxa to all remaining lineages from this species complex. *Tarentola neglecta *and *T. mindiae *are sister taxa to the *T. fascicularis*/*deserti *complex, and the split between these groups occurred around 7.04 (6.29-8.86) Mya. The phylogenetic relationships within the *T. fascicularis*/*deserti *complex remain unresolved, and with little geographic correspondence, although all North Algerian clades (Clades XIII, and XV) were clustered together, and represent a distinct lineage. The cladogenesis between the *T. mauritanica *complex and the clade formed by the *T. fascicularis*/*deserti *complex plus *T. mindiae *and *T. neglecta *took place around 8.69 (6.62-10.94) Mya, corresponding to the mid-late Miocene.

## Discussion

In light of the ages obtained for the node estimation, it is clear that the genealogy of the North African *Tarentola *dates back to the Miocene, with some speciation events predating most of the known paleogeographic occurrences that took place in the western Mediterranean. This is true regarding the early split of the ancestor of the lineage formed by *T. annularis, T. chazaliae *and *T. ephippiata*, and for the origin of *T. boehmei*. Interestingly, the split between the *T. mauritanica *complex and its sister clade formed by the ancestor of *T. neglecta, T. mindiae *and the *T. fascicularis/deserti *complex, was around 8.69 Mya (mid-late Miocene), which is coincident with the period of uplift of the Atlas Mountain chain [10]. The establishment of this geographic barrier may have separated the ancestor of the *T. mauritanica *complex to the western part of the Atlas, subject to a semiarid and sub-humid bioclimatic region, while the other major lineage could have been isolated eastwards, and confined to a more arid and Saharan typical habitat. Later, around 7.04 Mya the common ancestor of *T. neglecta *and *T. mindiae *separated from the *T. fascicularis/deserti *complex. The maintenance of *Tarentola *species adapted to a Saharan climate still occurs today, with *T. deserti, T. neglecta *and *T. mindiae *all occurring in drier regions than *T. mauritanica *[20,32,33]. The vicariant effect of the Moroccan Atlas Mountains has also been detected in other reptile species, such as *Agama impalearis *[11] and *Mauremys leprosa *[12].

The connection between the Mediterranean Sea and the Atlantic Ocean was interrupted during the late Miocene in a period that started 5.96 Mya and ceased 5.33 Mya, causing the Messinian Salinity Crisis [1-3]. This event led to the establishment of a land bridge between Africa and Europe and an opportunity for faunistic interchange, as reported for the spiny-footed lizard *Acanthodactylus erythrurus *[4], the snake *Natrix maura *[36], the skink *Chalcides bedriagae *[37] and the newts of the genus *Pleurodeles *[5]. Regarding the *T. mauritanica *complex, cladogenesis between Clade VI and the remaining lineages (5.88 Mya) matches very closely the closing of the Strait of Gibraltar, and is contemporary to the Messinian Salinity Crisis. According to our node age estimates, the separation between Clade IV (Central Morocco) and the ancestor of both clades II (Iberia) and III (Europe) occurred during the early Pliocene, around 4.14 Mya, after the opening of the Strait of Gibraltar, although the confidence interval could cover this period (up to 5.41 Mya). Therefore, considering the confidence interval of this node estimate, the colonization of the Iberian Peninsula by *Tarentola *from North Africa could have been during the Messinian Salinity Crisis, or after the opening of the Strait of Gibraltar during the early Pliocene. The crossing of the Strait of Gibraltar after it reopened has already been documented for the *Podarcis *wall lizards [38-40] and *Psammodromus algirus *[41].

Our results recover a well-supported mitochondrial phylogenetic tree for *Tarentola *from North Africa, adding considerably to what was previously known concerning this genus [17,19,26-30]. However, mitochondrial gene trees can be misleading particularly due to historical hybridization and introgression [e.g. [42]], and data from multiple unlinked loci should be used to infer phylogenetic history. Our nuclear phylogeny, while limited in resolving power, with several polytomies for the most recent nodes, did recover four of the six major lineages identified with the mtDNA alone.

Although, much debate has surrounded the validity of *T. angustimentalis *as a full species [21,26-30], our results from the nuclear DNA do support this taxon as an independent lineage within the *T. mauritanica *complex. Therefore, the recognition of *T. angustimentalis *implies that the *T. mauritanica *group is, in fact, a complex of several distinct forms that need to be recognized as full species. However, we suggest that elevation of independent lineages to specific status should only be done after an assessment of morphological variation, and detailed sampling to fully determine their distribution. Various recent studies have highlighted how taxonomic changes can be premature when cryptic diversity occurs, even if prior geographic sampling is extensive [e.g. [38]]. The primary objective of this work is not to make taxonomic revisions, but to assess the phylogenetic relationships within and between Mediterranean *Tarentola*.

According to the combined analysis, a monophyletic *T. deserti *was recovered, but this group renders *T. fascicularis *as a paraphyletic assemblage. It seems likely that *T. fascicularis *is actually a complex of lineages, some of which may deserve specific status. In order to unravel their evolutionary relationships, additional sampling and a morphological evaluation are required, especially across the western part of *T. fascicularis*' distribution range, and further identification of possible hybrids and contact zones. Regarding the recovered North Algerian lineages (XV and XIII), at least for clade XV there is already morphological evidence that this is, in fact, a distinct species (Julien Viglione, pers. comm.). If so, our phylogenetic data gives support to this as a monophyletic lineage.

## Conclusions

Mediterranean *Tarentola *are a complex of species complexes whose differentiation was initiated in the middle Miocene. The advent of multilocus approaches has, in general, caused researchers to move away from a strict reliance on gene trees, and it is now recognized that the signal from one gene tree is less important than the sum of signals across gene trees and loci [43]. Therefore, we support the phylogeny obtained by the combined data, which supports some already accepted relationships, but also indicates various novel ones:

(1) *T. annularis *and *T. ephippiata *are sister taxa, and *T. chazaliae *appears as a sister taxon to this pair of species. These three species differentiated from the remaining *Tarentola *during the Miocene, around 15.38 Mya.

(2) *T. boehmei *presents considerable genetic variation, and is sister taxon to all remaining North African lineages, comprised by the *T. mauritanica *complex, *T. fascicularis/deserti *complex, *T. neglecta*, and *T. mindiae*. The cladogenesis of *T. boehmei *occurred around 11.38 Mya.

(3) Regarding the *T. mauritanica *complex, Clade VI is sister taxon to all remaining lineages [26-30], whose differentiation took place around 5.88 Mya.

(4) A new lineage within the *T. mauritanica *complex was identified (Clade IV) from Central Morocco, sister taxon of Clades II and III.

(5) *T. angustimentalis *is recovered as a monophyletic lineage within the *T. mauritanica *complex.

(6) *T. neglecta *and *T. mindiae *form a sister clade to a complex of *T. deserti *and *T. fascicularis*, as opposed to the relationships recovered by Joger and Bshaenia [17]. The ancestor of both *T. neglecta *and *T. mindiae *got split from their sister taxa about 7.04 Mya.

(7) *T. fascicularis *is paraphyletic with respect to *T. deserti*.

(8) Two North Algerian clades (XV and XIII) are recovered, that are weakly supported as the sister taxa to the remaining specimens of the *T. fascicularis/deserti *complex.

(9) The separation between the *T. mauritanica *complex and the clade comprised by the ancestor of *T. neglecta, T. mindiae *and the *T. fascicularis/deserti *complex, occurred around 8.69 Mya (mid-late Miocene), which is coincident with the period of uplift of the Atlas Mountain chain.

This study has considerably refined the major groups that exist, and at least in the Iberian Peninsula and Northwestern Africa has defined their biogeographic patterns. The situation in Northeastern Africa remains more obscure. Detailed morphological and modelling approaches, combined with further sampling will be needed to ensure that taxonomic changes can be made in order to reflect the complex evolutionary history of the group.

## Methods

### DNA extraction, amplification and sequencing

Tissue from tail tip muscle was collected from each individual and preserved in 96% ethanol. Genomic DNA was extracted using the DNeasy Extraction Kit from Qiagen following the manufacturer's protocol. A total of 384 individuals were used, belonging to all known species of *Tarentola *in the Mediterranean Basin. Some of the individuals included in this study were already used in previous works [17,26-30,44,45]. Geographic location of each specimen is represented in Figure 2, and detailed information about locality and amplified genes in Table S1 from Additional file 2.

For all individuals the Polymerase Chain Reaction (PCR) amplification and sequencing of two mtDNA gene fragments, the 12SrRNA and 16SrRNA was performed using the primers 12Sa/12Sb and 16Sar/16Sbr from Kocher *et al. *[46] and Palumbi [47], respectively. PCR conditions were the same as those described in Harris *et al. *[48]. Four nuclear protein-coding gene fragments were also sequenced: the acetylcholinergic receptor M4 (ACM4), the melanocortin 1 receptor (MC1R), the phosducin (PDC), and the recombination activating gene 2 (RAG2). For amplification and sequencing of ACM4 the primers tg-F and tg-R published by Gamble *et al. *[24] were used. Regarding the MC1R fragment the primers MC1R_F and MC1R_R from Pinho *et al. *[49] were used, and primers PHOF2 and PHOR1 [50] for the amplification and sequencing of PDC. Amplification of ACM4, MC1R and PDC fragments were carried out in 25 μl volumes, containing 5.0 μl of 10x reaction Buffer, 2.0 mM of MgCl2, 0.5 mM each dNTP, 0.2 μM each primer, 1 U of Taq DNA polymerase (Invitrogen), and approximately 100 ng of template DNA. Finally, amplification and sequencing of the RAG2 gene fragment was performed using two sets of primers; 31FN.Venk/Lung.460R (amplification) and Lung.35F/Lung.320R (amplification and sequencing) published by Hoegg *et al. *[51]. PCR conditions were the same as described in Chiari *et al. *[52]. All amplified fragments were sequenced in a ABI3730XL automated sequencer.

The obtained sequences were imported into the software Geneious Pro v5.4.0 [53] where alignment was performed with MAFFT v6.814b [54] using the default parameters (auto algorithm; scoring matrix = 200 PAM/k = 2; gap open penalty = 1.53; and offset value = 0.123). All sequences generated in this study were submitted to GenBank with accession numbers ranging from JQ300539 to JQ301443. Detailed information on the individuals and sequences are described in the Table S1 from Additional file 2.

### Gene genealogies, and age estimates

Using the software ALTER [31] the mtDNA dataset was reduced to unique haplotypes, considering gaps as differences. Regarding the nuclear loci, heterozygous positions were coded with the corresponding ambiguity letter.

In order to determine the best fitting nucleotide model for each gene (mtDNA and nDNA), we used the software jModelTest v0.1.1 [55], under the Akaike Information Criterion [following [56]]. Maximum Likelihood (ML), and Bayesian Inference (BI) phylogenetic analyses were performed for both the concatenated mitochondrial and nuclear DNA datasets, and for all the loci (mtDNA+nDNA). ML analyses were conducted with the software RAxML v7.2.8 alpha [57], partitioning the dataset per locus. For all analyses 20 thorough ML searches were performed in order to obtain the best ML tree with support values, and thereafter 1000 bootstrap inferences. A majority rule consensus tree was generated using the software Phyutility [58]. BI was implemented with the program Mr.Bayes v3.1.2 [59] under a partitioned model (dataset divided into genes), and considering the model of nucleotide substitution estimated with jModelTest. The Bayesian posterior probabilities were estimated using a Metropolis-coupled Markov chain Monte Carlo sampling approach, and both runs started with random trees running for 10 × 10^6 ^generations, saving one tree every 100 generations producing 100,000 trees. Both convergence and appropriate sampling were confirmed by examining the standard deviation of the split frequencies between the two simultaneous runs and the Potential Scale Reduction Factor (PSRF) diagnostic. The first 25,000 trees of each run were included in the burn-in period and discarded. Next, a majority-rule consensus tree was generated from the remaining trees. In both phylogenetic analyses *Ptyodactylus hasselquistii *was used as outgroup.

The *T. boettgeri *group from the Canary and Selvages Islands has been used in previous analyses to provide a calibration point for estimates of the time of the cladogenetic events for the phylogeny of this genus [19,21], but the lack of available nuclear DNA sequences precludes its use in this study As an alternative, the substitution rate of the same mitochondrial region calculated for *Tarentola *was used for this purpose. Mean substitution rates and the standard error of the mean values for exactly the same 12S region as in the present study was extracted from a fully-calibrated phylogeny of *Tarentola *from the Canary islands [19,21]. This value was used as an informative prior in our divergence dating analysis. Specifically, we set a normal distribution prior for the ucld.mean parameter of the 12S partitions based on the result of the meanRate posterior (mean and standard error) of the calibration analyses of *Tarentola *(0.00891 ± 0.0000376 for the 12S).

We used BEAST v.1.6.1 [60] to estimate dates of the cladogenetic events from the concatenated dataset. The dataset comprised sequences from all six genes (nuclear genes unphased) using a phylogeny pruned arbitrarily to include one representative from each of the major lineages uncovered with the concatenated analysis (29 specimens in total). This method excludes closely related terminal taxa because the Yule tree prior (see below) does not include a model of coalescence, which can complicate rate estimation for closely related sequences [61]. Analyses were run four times for 5 × 10^7 ^generations with a sampling frequency of 10,000. Models and prior specifications applied were as follows (otherwise by default): GTR+I+G (12S, 16S), TN93 (ACM4), HKY+G (MC1R), HKY (PDC), TN93+G (RAG2); Relaxed Uncorrelated Lognormal Clock (estimate); Yule process of speciation; random starting tree; alpha Uniform (0, 10); yule.birthRate (0, 1000); ucld.mean of 12S Normal (initial value: 0.00827, mean: 0.00827, Stdev: 0.00162). Convergence for all model parameters was assessed by examining trace plots and histograms in Tracer v1.4 [62] after obtaining an effective sample size (ESS) > 200. The initial 10% of samples were discarded as burn-in. Runs were combined using LogCombiner, and maximum credibility trees with divergence time means and 95% highest probability densities (HPDs) were produced using Tree Annotator (both part of the BEAST package). Trees were visualized using FigTree v1.3.1 (available at http://tree.bio.ed.ac.uk/software/figtree).

## Authors' contributions

CR carried out the entire molecular laboratory work, analysed the data and drafted the manuscript. All authors participated in the conception and design of the study, collection of samples, writing and approval of the final manuscript.

## Supplementary Material

Additional file 1 **Figure S1**. Node age estimates among each mtDNA clade for the combined dataset, using the software BEAST v.1.6.1 and considering a relaxed uncorrelated lognormal clock and the Yule process of speciation as tree prior. Estimation of the used substitution rate is explained in the text. Click here for file

Additional file 2 **Table S1**. Table with information regarding the locality, and sequenced genes for all specimens used in this study. The specimens coded with a star have the same mitochondrial haplotype as individual *T. mauritanica*_400. Click here for file
