# Supplementary material for: Evolutionary history of the genus Tarentola (Gekkota: Phyllodactylidae) from the Mediterranean Basin, estimated using multilocus sequence data
Source: BMC Evol Biol. 2012 Jan 30;12:14. doi: 10.1186/1471-2148-12-14 (PMC3298722; doi:10.1186/1471-2148-12-14)
Supplement: Additional file 2 — Table S1. Table with information regarding the locality, and sequenced genes for all specimens used in this study. The specimens coded with a star have the same mitochondrial haplotype as individual T. mauritanica_400. [file 1471-2148-12-14-S2.DOCX]

| Species | **Code** | **Locality** | **Country** | **12S** | **16S** | **ACM4** | **MC1R** | **PDC** | **Rag2** |
| --- | --- | --- | --- | --- | --- | --- | --- | --- | --- |
| *Tarentola angustimentalis* | DB1318 | Nazaret-Teguise, Lanzarote | Spain | JQ300551 | JQ300945 | JQ301084 | JQ301141 | JQ301252 | JQ301424 |
|  | DB1326 | Yaiza, Lanzarote | Spain | JQ300705 | JQ300959 | JQ301062 | JQ301140 | JQ301218 | JQ301436 |
|  | DB1334 | Nazaret-Teguise, Lanzarote | Spain | JQ300598 | JQ300778 | JQ301017 | JQ301126 | JQ301235 | JQ301370 |
|  | DB1340 | La Rosa de Catalina, Fuerteventura | Spain | JQ300573 | JQ300852 | JQ301004 | JQ301187 | JQ301314 | JQ301355 |
|  | DB1353 | Yé, Lanzarote | Spain | JQ300736 | JQ300803 | JQ301082 | JQ301207 | JQ301292 | JQ301356 |
|  | DB1354 | Tefia, Fuerteventura | Spain | JQ300599 | JQ300918 | JQ301076 | JQ301121 | JQ301289 | JQ301409 |
|  | DB1357 | Tefia, Fuerteventura | Spain | JQ300581 | JQ300886 | * | * | * | * |
|  | DB1358 | Yaiza, Lanzarote | Spain | JQ300539 | JQ300993 | JQ301006 | JQ301183 | * | * |
|  | DB1365 | La Rosa de Catalina, Fuerteventura | Spain | JQ300671 | JQ300944 | JQ301078 | JQ301198 | * | * |
|  | DB1373 | Lazares, Fuerteventura | Spain | JQ300699 | JQ300947 | JQ301051 | JQ301180 | * | * |
|  | DB1374 | La Oliva, Fuerteventura | Spain | JQ300607 | JQ300820 | JQ301086 | JQ301122 | * | * |
|  | DB1389 | La Oliva, Fuerteventura | Spain | JQ300654 | JQ300991 | JQ301053 | JQ301199 | * | * |
|  | DB1402 | Yé, Lanzarote | Spain | JQ300640 | JQ301000 | JQ301069 | JQ301165 | * | * |
|  | DB1469 | Yé, Lanzarote | Spain | JQ300673 | JQ300773 | JQ301055 | JQ301168 | * | * |
|  | Ta20 | Fuerteventura | Spain | Harris et al. (2004b) | Harris et al. (2004b) | * | * | * | * |
| *Tarentola annularis* | SPM002386 | W de El Cairo, road to Bahariya | Egypt | JQ300566 | * | JQ301029 | JQ301125 | * | * |
| *Tarentola boehmei* | DB241 | Akka Ighane | Morocco | JQ300637 | JQ300878 | * | * | * | * |
|  | DB242 | Akka Ighane | Morocco | JQ300552 | JQ300952 | * | * | * | * |
|  | DB262 | Aït-Bekkou | Morocco | JQ300549 | JQ300903 | JQ301057 | JQ301176 | * | * |
|  | DB399 | 2km S of Zawyat Sidi Blal | Morocco | JQ300651 | JQ300898 | * | * | * | * |
|  | DB785 | Imi El Had | Morocco | JQ300608 | JQ300861 | * | * | * | * |
|  | DB795 | Tioulit | Morocco | JQ300557 | JQ300779 | * | * | * | * |
|  | DB859 | Abattekh | Morocco | JQ300656 | JQ300897 | * | JQ301146 | * | * |
|  | DB876 | Sidi Mohand Ou Sourou, Mirleft | Morocco | JQ300743 | JQ300869 | * | JQ301139 | * | * |
|  | DB878 | Idufkir, Sidi Ifni | Morocco | JQ300584 | JQ300922 | * | * | * | * |
|  | DB880 | Abattekh | Morocco | JQ300652 | JQ300781 | * | * | * | * |
|  | DB1411 | Gorges near Guelmin | Morocco | JQ300582 | JQ300782 | JQ301087 | JQ301185 | * | * |
|  | DB1604 | Foum Zguid | Morocco | JQ300748 | JQ300799 | * | * | * | * |
|  | DB1864 | Aouinet Torkoz | Morocco | Ceacero et al. (2010) | JQ300948 | JQ301089 | JQ301191 | * | * |
|  | DB9000 | 0.5 Km N. of Bou-Azzer | Morocco | JQ300668 | JQ300817 | JQ301096 | JQ301174 | * | * |
|  | DB11024 | Near Oed Iriri | Morocco | JQ300692 | JQ300824 | * | * | * | * |
|  | SC64 | Akka Ighane | Morocco | Harris et al. (2004b) | Harris et al. (2004b) | * | * | * | * |
| *Tarentola chazaliae* | DB863 | Abattekh | Morocco | JQ300722 | JQ300955 | JQ301036 | JQ301166 | * | * |
| *Tarentola chazaliae* | DB884 | Tafnidilt, Tan-Tan | Morocco | JQ300542 | JQ300797 | JQ301042 | JQ301204 | * | * |
| *Tarentola deserti* | DB325 | Merzouga (house) | Morocco | JQ300658 | JQ300921 | JQ301005 | JQ301134 | * | * |
|  | DB326 | Merzouga | Morocco | JQ300727 | JQ300920 | * | * | * | * |
|  | DB327 | Merzouga | Morocco | JQ300564 | JQ300971 | * | * | * | * |
|  | DB363 | Road between Messaad and Ain Kheneg ed Defia | Algeria | JQ300655 | JQ300864 | JQ301034 | JQ301202 | * | * |
|  | DB467 | Guemar, El Oued | Algeria | JQ300679 | JQ300848 | JQ301097 | JQ301197 | * | * |
|  | DB473 | Guemar, El Oued | Algeria | JQ300766 | JQ300835 | JQ301052 | JQ301171 | * | * |
|  | DB480 | Taibet, Touggourt | Algeria | JQ300708 | JQ300911 | JQ301044 | JQ301189 | * | * |
|  | DB481 | El Goléa, El Goléa (Sahara) | Algeria | JQ300628 | JQ300990 | JQ301016 | JQ301104 | * | * |
|  | DB488 | El Ateuf, Ghardaia (Sahara) | Algeria | JQ300579 | JQ300896 | * | * | * | * |
|  | DB1262 | Road between Messaad and Ain Kheneg ed Defia | Algeria | JQ300754 | JQ300967 | JQ301049 | JQ301107 | JQ301217 | JQ301363 |
|  | DB3124 |  | Morocco | JQ300718 | JQ300868 | * | * | * | * |
|  | DB3178 |  | Morocco | JQ300696 | JQ300996 | * | * | * | * |
|  | DB3278 |  | Morocco | JQ300723 | JQ300866 | JQ301056 | JQ301179 | * | * |
|  | DB8998 | 7 Km South of Rizane | Morocco | JQ300725 | JQ300880 | JQ301066 | JQ301170 | * | * |
|  | DB9005 | Rissani | Morocco | JQ300625 | JQ300917 | * | * | * | * |
|  | DB9006 | Rissani | Morocco | JQ300647 | JQ300977 | * | * | * | * |
|  | DB9007 | Rissani | Morocco | JQ300562 | JQ300857 | * | * | * | * |
|  | DB9011 | Erfoud area | Morocco | JQ300586 | JQ300934 | * | * | * | * |
|  | DB9015 | 7 Km South of Rizane | Morocco | JQ300615 | JQ300925 | JQ301033 | JQ301154 | * | * |
|  | DB9016 | 7 Km South of Rizane | Morocco | JQ300548 | JQ300930 | * | * | * | * |
|  | SC62 | Erfoud | Morocco | Carranza et al. (2002) | Harris et al. (2004b) | * | * | * | * |
|  | Td55 | 5 km N Arfoud | Morocco | Harris et al. (2004b) | Harris et al. (2004b) | * | * | * | * |
| *Tarentola ephippiata* | SPM000387 | Smara | Western Sahara | JQ300681 | JQ300845 | * | * | * | * |
| *Tarentola ephippiata* | SPM003398 | 25Km W of Smara | Western Sahara | JQ300746 | JQ300846 | JQ301088 | JQ301123 | * | * |
| *Tarentola fascicularis* | DB2143 | Tulmaythah | Libya | JQ300653 | JQ300780 | * | * | * | * |
|  | DB2146 | Aen Alzeana | Libya | JQ300695 | JQ300876 | * | * | * | * |
|  | DB2152 | 120 km NE towards Bengazi | Libya | JQ300580 | JQ300828 | * | * | * | * |
|  | DB2155 | Al Mabne | Libya | JQ300630 | JQ300838 | * | * | * | * |
|  | DB2156 | Detj oasis | Libya | JQ300646 | JQ300941 | * | * | * | * |
|  | DB2158 | Tulmaythah | Libya | JQ300614 | JQ300968 | * | * | * | * |
|  | DB2159 | Detj oasis | Libya | JQ300757 | JQ300829 | * | * | * | * |
|  | DB2160 | Senebat Lauela | Libya | JQ300678 | JQ300889 | * | * | * | * |
|  | DB2163 | Gadamesh | Libya | JQ300664 | JQ300891 | JQ301025 | JQ301201 | * | * |
|  | DB2165 | Aen Alzeana | Libya | JQ300572 | JQ300890 | * | * | * | * |
|  | DB2168 | Maquis between Gandula and Al Beida | Libya | JQ300544 | JQ300982 | * | * | * | * |
|  | DB3132 | Conigli islet | Italy | JQ300769 | JQ300879 | * | * | * | * |
|  | DB5932 | The international coastal road, 11km | Egypt | JQ300760 | JQ300791 | * | * | * | * |
|  | DB5946 | 3 km E of Zawyet el Hawale | Egypt | JQ300702 | JQ300961 | * | * | * | * |
|  | Tm21 | Libya/Egypt border | Libya | Harris et al. (2004b) | Harris et al. (2004b) | Rato et al. (2010) | Rato et al. (2010) | * | * |
|  | Tm22 | Maquis | Libya | Harris et al. (2004b) | Harris et al. (2004b) | * | * | * | * |
|  | Tm23 | Om Arazam | Libya | Harris et al. (2004b) | Harris et al. (2004b) | * | * | * | * |
|  | Tm26 | Tobruk | Libya | Harris et al. (2004b) | Harris et al. (2004b) | Rato et al. (2010) | Rato et al. (2010) | * | * |
| *Tarentola f. wolfgangi* | IB47 | Bou Hedma National Park | Tunisia | Joger and Bshaenia (2010) | * | * | * | * | * |
| *Tarentola mauritanica* | DB177***** | Zafra | Spain | Rato et al. (2010) | Rato et al. (2010) | * | * | * | * |
|  | DB190 | 31 km Azni | Morocco | Rato et al. (2010) | Rato et al. (2010) | * | * | * | * |
|  | DB214***** | Guelta Safra | Tunisia | Rato et al. (2010) | Rato et al. (2010) | Rato et al. (2010) | Rato et al. (2010) | * | * |
|  | DB215***** | Guelta Safra | Tunisia | Rato et al. (2010) | Rato et al. (2010) | Rato et al. (2010) | Rato et al. (2010) | * | * |
|  | DB216 | Guelta Safra | Tunisia | Rato et al. (2010) | Rato et al. (2010) | Rato et al. (2010) | Rato et al. (2010) | * | * |
|  | DB218***** | Guelta Safra | Tunisia | Rato et al. (2010) | Rato et al. (2010) | JQ301023 | JQ301106 | * | * |
|  | DB265 | Malcata | Portugal | Rato et al. (2010) | Rato et al. (2010) | * | * | * | * |
|  | DB267***** | Ceuta | Spain | Rato et al. (2010) | Rato et al. (2010) | * | * | * | * |
|  | DB270***** | Alboran | Spain | Rato et al. (2010) | Rato et al. (2010) | * | * | * | * |
|  | DB272***** | Adra, Almería | Spain | Rato et al. (2010) | Rato et al. (2010) | Rato et al. (2010) | Rato et al. (2010) | * | * |
|  | DB273***** | Melilla | Spain | Rato et al. (2010) | Rato et al. (2010) | Rato et al. (2010) | Rato et al. (2010) | * | * |
|  | DB292***** | Cantaritas, Sevilla | Spain | Rato et al. (2010) | Rato et al. (2010) | * | * | * | * |
|  | DB311 | Michliffen | Morocco | Rato et al. (2010) | Rato et al. (2010) | Rato et al. (2010) | Rato et al. (2010) | * | * |
|  | DB313 | Marrakech | Morocco | JQ300742 | JQ300913 | JQ301100 | JQ301145 | * | * |
|  | DB314 | Marrakech | Morocco | JQ300680 | JQ300966 | * | * | * | * |
|  | DB317 | Moulay Idris | Morocco | Rato et al. (2010) | Rato et al. (2010) | JQ301067 | JQ301137 | * | * |
|  | DB318 | Midelt | Morocco | Rato et al. (2010) | Rato et al. (2010) | * | * | * | * |
|  | DB320 | Essaouira | Morocco | Rato et al. (2010) | Rato et al. (2010) | * | * | * | * |
|  | DB321 | Essaouira | Morocco | Rato et al. (2010) | Rato et al. (2010) | * | * | * | * |
|  | DB323***** | Moulay Bousselham | Morocco | Rato et al. (2010) | Rato et al. (2010) | * | * | * | * |
|  | DB328 | Moulay Bousselham | Morocco | Rato et al. (2010) | Rato et al. (2010) | Rato et al. (2010) | Rato et al. (2010) | * | * |
|  | DB329 | Tirmest | Morocco | Rato et al. (2010) | Rato et al. (2010) | * | * | * | * |
|  | DB351***** | Cape Serrat | Tunisia | JQ300667 | JQ300912 | * | * | * | * |
|  | DB365***** | Barrosa, Cádiz | Spain | Rato et al. (2010) | Rato et al. (2010) | * | * | * | * |
|  | DB367 | Galera, Granada | Spain | Rato et al. (2010) | Rato et al. (2010) | * | * | * | * |
|  | DB368 | Galera, Granada | Spain | Rato et al. (2010) | Rato et al. (2010) | * | * | * | * |
|  | DB369***** | Barrosa, Cádiz | Spain | Rato et al. (2010) | Rato et al. (2010) | * | * | * | * |
|  | DB373 | Talassetam | Morocco | Rato et al. (2010) | Rato et al. (2010) | * | * | * | * |
|  | DB374 | Talassetam | Morocco | Rato et al. (2010) | Rato et al. (2010) | * | * | * | * |
|  | DB377 | Granada | Spain | Rato et al. (2010) | Rato et al. (2010) | * | * | * | * |
|  | DB379 | Benadalid, Málaga | Spain | Rato et al. (2010) | Rato et al. (2010) | * | * | * | * |
|  | DB381 | Galera, Granada | Spain | Rato et al. (2010) | Rato et al. (2010) | * | * | * | * |
|  | DB394***** | Palace Doñana National Park, Huelva | Spain | Rato et al. (2010) | Rato et al. (2010) | * | * | * | * |
|  | DB395***** | Matalascanas, Huelva | Spain | Rato et al. (2010) | Rato et al. (2010) | Rato et al. (2010) | Rato et al. (2010) | * | * |
|  | DB398 | 10km S of Touama | Morocco | Rato et al. (2010) | Rato et al. (2010) | JQ301072 | JQ301143 | * | * |
|  | DB401 | Torcal de Antequera, Málaga | Spain | Rato et al. (2010) | Rato et al. (2010) | Rato et al. (2010) | Rato et al. (2010) | * | * |
|  | DB402***** | NW Mazagón, Huelva | Spain | Rato et al. (2010) | Rato et al. (2010) | * | * | * | * |
|  | DB403***** | Santa Ana, Jaén | Spain | Rato et al. (2010) | Rato et al. (2010) | Rato et al. (2010) | Rato et al. (2010) | * | * |
|  | DB404***** | Alcalá la Real, Jaén | Spain | Rato et al. (2010) | Rato et al. (2010) | * | * | * | * |
|  | DB405***** | Beja | Portugal | Rato et al. (2010) | Rato et al. (2010) | * | * | * | * |
|  | DB406***** | Doñana, Huelva | Spain | Rato et al. (2010) | Rato et al. (2010) | * | * | * | * |
|  | DB407 | Santuario de la Virgen de la Cabeza, Andújar, Jaén | Spain | Rato et al. (2010) | Rato et al. (2010) | * | * | * | * |
|  | DB408***** | Lagunas de Moguer | Spain | Rato et al. (2010) | Rato et al. (2010) | * | * | * | * |
|  | DB409***** | Playa de la Ballena | Spain | Rato et al. (2010) | Rato et al. (2010) | * | * | * | * |
|  | DB410***** | Playa de la Vibora, Málaga | Spain | Rato et al. (2010) | Rato et al. (2010) | * | * | * | * |
|  | DB411 | Ocaña, Cuenca | Spain | Rato et al. (2010) | Rato et al. (2010) | * | * | * | * |
|  | DB412 | Cabañeros National Park, Ciudad Real | Spain | Rato et al. (2010) | Rato et al. (2010) | Rato et al. (2010) | Rato et al. (2010) | * | * |
|  | DB413 | Valencia del Ventoso, Badajoz | Spain | Rato et al. (2010) | Rato et al. (2010) | * | * | * | * |
|  | DB414 | Cornalvo Natural Park, Badajoz | Spain | Rato et al. (2010) | Rato et al. (2010) | * | * | * | * |
|  | DB415 | Cabañeros National Park, Ciudad Real | Spain | Rato et al. (2010) | Rato et al. (2010) | * | * | * | * |
|  | DB416 | 7kms N of Castelo de Vide | Portugal | Rato et al. (2010) | Rato et al. (2010) | * | * | * | * |
|  | DB417 | Lagunas de Ruidera, Albacete | Spain | Rato et al. (2010) | Rato et al. (2010) | * | * | * | * |
|  | DB420***** | La Roda (Estación RENFE) | Spain | Rato et al. (2010) | Rato et al. (2010) | * | * | * | * |
|  | DB423***** | Pantano cerca de Thibar | Tunisia | Rato et al. (2010) | Rato et al. (2010) | * | * | * | * |
|  | DB424***** | PN Beja | Tunisia | Rato et al. (2010) | Rato et al. (2010) | * | * | * | * |
|  | DB425***** | Tabarka | Tunisia | Rato et al. (2010) | Rato et al. (2010) | * | * | * | * |
|  | DB426***** | Chemtou | Tunisia | Rato et al. (2010) | Rato et al. (2010) | * | * | * | * |
|  | DB474 | Theniet el had National Park | Algeria | JQ300558 | JQ300783 | JQ301063 | JQ301206 | * | * |
|  | DB476***** | Tizi n'Tléta, Tizi-Ouzou | Algeria | JQ300674 | JQ300875 | * | * | * | * |
|  | DB479***** | Djurdjura National Park | Algeria | JQ300618 | JQ300946 | JQ301028 | JQ301150 | * | * |
|  | DB494***** | Chréa National Park | Algeria | JQ300635 | JQ300788 | * | * | * | * |
|  | DB496***** | Djurdjura National Park | Algeria | JQ300660 | JQ300901 | JQ301050 | JQ301115 | * | * |
|  | DB526***** | Capri Island | Italy | JQ300693 | JQ300831 | JQ301065 | JQ301105 | * | * |
|  | DB687***** | Chiaiolella Port, Procida Island | Italy | JQ300623 | JQ300795 | * | * | * | * |
|  | DB688***** | Via Tortora, Procida Island | Italy | JQ300716 | JQ300842 | JQ301071 | JQ301164 | * | * |
|  | DB696***** | Pizzaco, Procida Island | Italy | JQ300704 | JQ300787 | JQ301079 | JQ301167 | * | * |
|  | DB703***** | Barano, Ischia Island | Italy | JQ300709 | JQ300794 | * | * | * | * |
|  | DB720***** | Ischia Port, Ischia Island | Italy | JQ300719 | JQ300796 | JQ301002 | JQ301162 | * | * |
|  | DB721***** | Ischia Port, Ischia Island | Italy | JQ300645 | JQ300883 | * | * | * | * |
|  | DB722***** | Barano, Ischia Island | Italy | JQ300605 | JQ300923 | * | * | * | * |
|  | DB724***** | Barano, Ischia Island | Italy | JQ300627 | JQ300833 | JQ301094 | JQ301117 | * | * |
|  | DB756***** | Vico Equense, Cimitero, Campania Continental | Italy | JQ300570 | JQ300973 | JQ301041 | JQ301186 | * | * |
|  | DB766***** | Pizzaco, Procida Island | Italy | JQ300583 | JQ300867 | * | * | * | * |
|  | DB780 | Agdal Riyad, Rabat | Morocco | JQ300750 | JQ300994 | JQ301101 | JQ301119 | * | * |
|  | DB911 | Semda, Soukkane | Morocco | JQ300670 | JQ300983 | JQ301103 | JQ301203 | * | * |
|  | DB924 | Chouga, Sidi el Bettach | Morocco | JQ300631 | JQ300975 | * | * | * | * |
|  | DB925 | Semda, Soukkane | Morocco | JQ300677 | JQ300858 | JQ301092 | JQ301129 | * | * |
|  | DB933 | Chouga, Sidi el Bettach | Morocco | JQ300713 | JQ300942 | JQ301003 | JQ301173 | * | * |
|  | DB934 | Chouga, Sidi el Bettach | Morocco | JQ300733 | JQ300815 | JQ301091 | JQ301130 | * | * |
|  | DB935 | Chouga, Sidi el Bettach | Morocco | JQ300587 | JQ300826 | * | * | * | * |
|  | DB1207***** | Ciudad Rodrigo | Spain | JQ300710 | JQ300976 | JQ301022 | JQ301161 | * | * |
|  | DB1212***** | Ciudad Rodrigo | Spain | JQ300717 | JQ300902 | * | * | * | * |
|  | DB1236 | Caravaca de la Cruz | Spain | JQ300714 | JQ300822 | * | * | * | * |
|  | DB1246***** | Villanueva de Córdoba | Spain | JQ300720 | JQ300816 | JQ301031 | JQ301124 | * | * |
|  | DB1293 | Embalse de Camarillas | Spain | JQ300767 | JQ300836 | JQ301070 | JQ301108 | * | * |
|  | DB1294 | Riopar Viejo | Spain | JQ300703 | JQ300908 | JQ301001 | JQ301127 | * | * |
|  | DB1295 | Cañada del Provencio | Spain | JQ300669 | JQ300907 | * | * | * | * |
|  | DB1397 | Near Sidi-Chikér | Morocco | JQ300559 | JQ300771 | * | * | * | * |
|  | DB1400 | Near Sidi-Chikér | Morocco | JQ300675 | JQ300957 | * | * | * | * |
|  | DB1448 | Argana-Jebel Oulime North | Morocco | JQ300690 | JQ300940 | * | * | * | * |
|  | DB1519***** | Asilah | Morocco | JQ300611 | JQ300932 | * | * | * | * |
|  | DB1571***** | Bou Ficha, Zaghouan | Tunisia | JQ300732 | JQ300950 | * | * | * | * |
|  | DB1666***** | Montpellier | France | JQ300601 | JQ300981 | * | * | * | * |
|  | DB1667***** | Montpellier | France | JQ300588 | JQ300844 | * | * | * | * |
|  | DB1774 | Peña del Olivar, Jaén | Spain | JQ300764 | JQ300825 | * | * | * | * |
|  | DB1786 | Cortijo de los Petrolos, Jaén | Spain | JQ300629 | JQ300989 | * | * | * | * |
|  | DB1801 | Cortijo de Moralejos, Jaén | Spain | JQ300577 | JQ300936 | * | * | * | * |
|  | DB1880 | Albacete | Spain | JQ300569 | JQ300798 | * | * | * | * |
|  | DB2545 | Argana-Jebel Oulime North | Morocco | JQ300560 | JQ300960 | * | * | * | * |
|  | DB2563 | Skour-Rehamna | Morocco | JQ300554 | JQ300819 | JQ301045 | JQ301112 | * | * |
|  | DB2635 | 2 km before Ait-barka | Morocco | JQ300657 | JQ300821 | * | * | * | * |
|  | DB2636 | Tasguint (Taurodant) | Morocco | JQ300547 | JQ300843 | * | * | * | * |
|  | DB2818 | Area recreativa de los estrechos, Jaén | Spain | JQ300751 | JQ300997 | * | * | * | * |
|  | DB2886 | Peña del Olivar, Jaén | Spain | JQ300672 | JQ300793 | * | * | * | * |
|  | DB3131***** | iMarina di Cerveteri, Latium | Italy | JQ300729 | JQ300830 | * | * | * | * |
|  | DB3142***** | Monte S. Elia, Apulia | Italy | JQ300659 | JQ300802 | * | * | * | * |
|  | DB3144***** | Monte S. Elia, Apulia | Italy | JQ300685 | JQ300785 | * | * | * | * |
|  | DB3145***** | Monte S. Elia, Apulia | Italy | JQ300728 | JQ300895 | JQ301098 | JQ301148 | * | * |
|  | DB3146***** | Monte S. Elia, Apulia | Italy | JQ300632 | JQ300893 | * | * | * | * |
|  | DB3148***** | Monte S. Elia, Apulia | Italy | JQ300747 | JQ300806 | JQ301008 | JQ301132 | * | * |
|  | DB3152***** | Gruxi Lillius, Sardinia | Italy | JQ300602 | JQ300849 | JQ301014 | JQ301131 | * | * |
|  | DB3170***** | Infernetto, Latium | Italy | JQ300591 | JQ300935 | * | * | * | * |
|  | DB3176***** | Infernetto, Latium | Italy | JQ300731 | JQ300881 | JQ301024 | JQ301110 | * | * |
|  | DB3179***** | Infernetto, Latium | Italy | JQ300626 | JQ300974 | JQ301085 | JQ301177 | * | * |
|  | DB3180***** | Monte S. Elia, Apulia | Italy | JQ300589 | JQ300963 | * | * | * | * |
|  | DB3182***** | Gesturi, Sardinia | Italy | JQ300650 | JQ300818 | JQ301090 | JQ301138 | * | * |
|  | DB3231***** | Zadar | Croatia | JQ300616 | JQ300777 | JQ301060 | JQ301116 | * | * |
|  | DB3232***** | Zadar | Croatia | JQ300694 | JQ300992 | * | * | * | * |
|  | DB3233***** | Hvar | Croatia | JQ300609 | JQ300877 | * | * | * | * |
|  | DB3234***** | Zadar | Croatia | JQ300617 | JQ300926 | * | * | * | * |
|  | DB3235***** | Hvar | Croatia | JQ300740 | JQ300888 | JQ301030 | JQ301195 | * | * |
|  | DB3236***** | Hvar | Croatia | JQ300606 | JQ300884 | * | * | * | * |
|  | DB3238***** | Zadar | Croatia | JQ300642 | JQ300927 | * | * | * | * |
|  | DB3239***** | Hvar | Croatia | JQ300730 | JQ300999 | * | * | * | * |
|  | DB3832 | Redonda island, Mar Menor, Murcia | Spain | JQ300735 | JQ300988 | * | * | * | * |
|  | DB3834 | Barrón island, Mar Menor, Murcia | Spain | JQ300561 | JQ300812 | * | * | * | * |
|  | DB3838 | Grosa island, Murcia | Spain | JQ300739 | JQ300972 | * | * | * | * |
|  | DB3839 | Grosa island, Murcia | Spain | JQ300593 | JQ300792 | * | * | * | * |
|  | DB3843 | Sujeto island, Mar Menor, Murcia | Spain | JQ300726 | JQ300900 | * | * | * | * |
|  | DB3844 | Sujeto island, Mar Menor, Murcia | Spain | JQ300715 | JQ300810 | * | * | * | * |
|  | DB3846 | Redonda island, Mar Menor, Murcia | Spain | JQ300567 | JQ300980 | * | * | * | * |
|  | DB3847 | Tabarca island, Alicante | Spain | JQ300603 | JQ300962 | JQ301064 | JQ301149 | * | * |
|  | DB3848 | Tabarca island, Alicante | Spain | JQ300706 | JQ300865 | JQ301073 | JQ301135 | * | * |
|  | DB3853 | Rambla del Cañar, Murcia | Spain | JQ300540 | JQ300931 | JQ301010 | JQ301155 | * | * |
|  | DB5057 | Ait Ou Ba Allal | Morocco | JQ300565 | JQ300919 | * | * | * | * |
|  | DB5065 | Talzemt | Morocco | JQ300761 | JQ300784 | JQ301059 | JQ301200 | * | * |
|  | DB5113 | Talzemt | Morocco | JQ300585 | JQ300954 | * | * | * | * |
|  | DB5142***** | Sta. Barbara, Amposta, Tarragona | Spain | JQ300741 | JQ300987 | * | * | * | * |
|  | DB9076 | Jbelet | Morocco | JQ300555 | JQ300928 | * | * | * | * |
|  | DB9104***** | Bardari, Calabria | Italy | JQ300594 | JQ300809 | * | * | * | * |
|  | DB9107***** | Capo Colonna, Calabria | Italy | JQ300545 | JQ300850 | JQ301080 | JQ301133 | * | * |
|  | DB9111***** | Zambrone, Calabria | Italy | JQ300641 | JQ300887 | * | * | * | * |
|  | DB9112***** | Maiori, Campania | Italy | JQ300633 | JQ300986 | JQ301009 | JQ301192 | * | * |
|  | DB9113***** | Zambrone, Calabria | Italy | JQ300661 | JQ300970 | JQ301011 | JQ301109 | * | * |
|  | DB9115***** | Bardari, Calabria | Italy | JQ300639 | JQ300851 | JQ301068 | JQ301194 | * | * |
|  | DB9116* | 30Km From Amendolara, Calabria | Italy | JQ300749 | JQ300804 | * | * | * | * |
|  | DB11003 | Marrakech University | Morocco | JQ300578 | JQ300862 | * | * | * | * |
|  | DB11004 | Marrakech University | Morocco | JQ300597 | JQ300914 | JQ301015 | JQ301118 | * | * |
|  | DB11007 | Sidi-Moktar | Morocco | JQ300624 | JQ300786 | * | * | * | * |
|  | DB11008 | Marrakech University | Morocco | JQ300662 | JQ300906 | JQ301018 | JQ301193 | * | * |
|  | DB11009 | Anezal | Morocco | JQ300595 | JQ300859 | JQ301099 | JQ301147 | * | * |
|  | DB11011***** | Maiori, Campania | Italy | JQ300665 | JQ300894 | * | * | * | * |
|  | DB11013 | Marrakech University | Morocco | JQ300612 | JQ300929 | JQ301075 | JQ301142 | * | * |
|  | DB11015 | Jbelet | Morocco | JQ300765 | JQ300808 | * | * | * | * |
|  | DB11019 | Jbelet | Morocco | JQ300744 | JQ300873 | JQ301047 | JQ301169 | * | * |
|  | DB11021 | Jbelet | Morocco | JQ300698 | JQ300834 | * | * | * | * |
|  | DB11022 | Ain-el-Hjar | Morocco | JQ300541 | JQ300860 | JQ301027 | JQ301175 | * | * |
|  | DB11029 | Jbelet | Morocco | JQ300756 | JQ300789 | * | * | * | * |
|  | DB11035 | Sidi-Moktar | Morocco | JQ300636 | JQ300871 | * | * | * | * |
|  | DB11042 | Marrakech University | Morocco | JQ300686 | JQ300854 | * | * | * | * |
|  | DB11091***** | Cortes, Corsica Island | France | JQ300563 | JQ300951 | * | * | * | * |
|  | DB11097***** | Near Ponte Leccia, Corsica Island | France | JQ300620 | JQ300965 | * | * | * | * |
|  | DB11100***** | Near Nardo, Puglia | Italy | JQ300752 | JQ300885 | * | * | * | * |
|  | DB11105***** | Near Nardo, Puglia | Italy | JQ300758 | JQ300774 | JQ301039 | JQ301190 | * | * |
|  | 400 | Madeira island | Portugal | Jesus (2008) | Jesus (2008) | * | * | * | * |
|  | PS401***** | Porto Santo island | Portugal | Jesus (2008) | Jesus (2008) | * | * | * | * |
|  | SC63 | Abdelmaleh Rahmd | Algeria | Harris et al. (2004b) | Harris et al. (2004b) | * | * | * | * |
|  | Tm1***** | Zahara Atunes | Spain | Harris et al. (2004b) | Harris et al. (2004b) | * | * | * | * |
|  | Tm2***** | Zahara Atunes | Spain | Harris et al. (2004b) | Harris et al. (2004b) | * | * | * | * |
|  | Tm3***** | Barcelona | Spain | Harris et al. (2004b) | Harris et al. (2004b) | Rato et al. (2010) | Rato et al. (2010) | * | * |
|  | Tm4***** | Barrosa, Cádiz | Spain | Harris et al. (2004b) | Harris et al. (2004b) | Rato et al. (2010) | Rato et al. (2010) | * | * |
|  | Tm5***** | Barrosa, Cádiz | Spain | Harris et al. (2004b) | Harris et al. (2004b) | * | * | * | * |
|  | Tm6***** | Monte Clerigo | Portugal | Harris et al. (2004b) | Harris et al. (2004b) | Rato et al. (2010) | Rato et al. (2010) | * | * |
|  | Tm7***** | Monte Clerigo | Portugal | Harris et al. (2004b) | Harris et al. (2004b) | * | * | * | * |
|  | Tm10 | Bab Taza | Morocco | Harris et al. (2004b) | Harris et al. (2004b) | * | * | * | * |
|  | Tm11 | Road to Tizi n`Test | Morocco | Harris et al. (2004b) | Harris et al. (2004b) | Rato et al. (2010) | Rato et al. (2010) | * | * |
|  | Tm12***** | Jabal Ziblah | Tunisia | Harris et al. (2004b) | Harris et al. (2004b) | * | * | * | * |
|  | Tm13***** | Kebir | Tunisia | Harris et al. (2004b) | Harris et al. (2004b) | Rato et al. (2010) | Rato et al. (2010) | JQ301310 | Rato et al. (2010) |
|  | Tm14***** | Tabarka | Tunisia | Harris et al. (2004b) | Harris et al. (2004b) | * | * | * | * |
|  | Tm15***** | Napoli | Italy | Harris et al. (2004b) | Harris et al. (2004b) | * | * | * | * |
|  | Tm16 | Taza | Morocco | Harris et al. (2004b) | Harris et al. (2004b) | JQ301040 | JQ301128 | * | * |
|  | Tm17***** | Madeira | Portugal | Harris et al. (2004b) | Harris et al. (2004b) | * | * | * | * |
|  | Tm24***** | Crete | Greece | Harris et al. (2004b) | Harris et al. (2004b) | * | * | * | * |
|  | Tm27***** | Zafra | Spain | Harris et al. (2004a) | Harris et al. (2004a) | Rato et al. (2010) | Rato et al. (2010) | * | * |
|  | Tm29***** | Zafra | Spain | Harris et al. (2004a) | Harris et al. (2004a) | Rato et al. (2010) | Rato et al. (2010) | * | * |
|  | Tm30***** | Huelva | Spain | Harris et al. (2004a) | Harris et al. (2004a) | Rato et al. (2010) | Rato et al. (2010) | * | * |
|  | Tm32 | Al Jadida | Morocco | Harris et al. (2004b) | Harris et al. (2004b) | * | * | * | * |
|  | Tm33 | Al Jadida | Morocco | Harris et al. (2004a) | Harris et al. (2004a) | * | * | * | * |
|  | Tm34 | Al Jadida | Morocco | Harris et al. (2004a) | Harris et al. (2004a) | * | * | * | * |
|  | Tm35 | Oulad Brahim | Morocco | Harris et al. (2004b) | Harris et al. (2004b) | * | * | * | * |
|  | Tm36 | Oulad Brahim | Morocco | Harris et al. (2004a) | Harris et al. (2004a) | * | * | * | * |
|  | Tm37 | Oulad Brahim | Morocco | Harris et al. (2004a) | Harris et al. (2004a) | * | * | * | * |
|  | Tm38 | Oulad Brahim | Morocco | Harris et al. (2004a) | Harris et al. (2004a) | * | * | * | * |
|  | Tm39 | Oulad Brahim | Morocco | Harris et al. (2004a) | Harris et al. (2004a) | * | * | * | * |
|  | Tm40 | Oulad Brahim | Morocco | Harris et al. (2004a) | Harris et al. (2004a) | * | * | * | * |
|  | Tm42 | 31 km Azni | Morocco | Harris et al. (2004a) | Harris et al. (2004a) | * | * | * | * |
|  | Tm43 | Aza | Morocco | Harris et al. (2004a) | Harris et al. (2004a) | Rato et al. (2010) | Rato et al. (2010) | * | * |
|  | Tm44 | Aza | Morocco | Harris et al. (2004a) | Harris et al. (2004a) | * | * | * | * |
|  | Tm45 | 10 km Azar | Morocco | Harris et al. (2004a) | Harris et al. (2004a) | * | * | * | * |
|  | Tm46 | 10 km Azar | Morocco | Harris et al. (2004a) | Harris et al. (2004a) | * | * | * | * |
|  | Tm48 | Agadir-Tizni | Morocco | Harris et al. (2004a) | Harris et al. (2004a) | * | * | * | * |
|  | Tm49 | Agadir-Tizni | Morocco | Harris et al. (2004a) | Harris et al. (2004a) | Rato et al. (2010) | Rato et al. (2010) | * | * |
|  | Tm50 | Massa | Morocco | Harris et al. (2004a) | Harris et al. (2004a) | Rato et al. (2010) | Rato et al. (2010) | * | * |
|  | Tm51 | Massa | Morocco | Harris et al. (2004b) | Harris et al. (2004b) | * | * | * | * |
|  | Tm52 | Merght | Morocco | Harris et al. (2004b) | Harris et al. (2004b) | * | * | * | * |
|  | Tm53 | Guelmine | Morocco | Harris et al. (2004a) | Harris et al. (2004a) | * | * | * | * |
|  | Tm54 | Argana | Morocco | Harris et al. (2004a) | Harris et al. (2004a) | * | * | * | * |
|  | Tm56 | Taza | Morocco | Harris et al. (2004a) | Harris et al. (2004a) | * | * | * | * |
|  | Tm57 | Taza | Morocco | Harris et al. (2004a) | Harris et al. (2004a) | * | * | * | * |
|  | Tm58***** | Assilah | Morocco | Harris et al. (2004a) | Harris et al. (2004a) | * | * | * | * |
|  | Tm59***** | Lithica, Menorca | Spain | Harris et al. (2004b) | Harris et al. (2004b) | * | * | * | * |
|  | Tm60***** | Lithica, Menorca | Spain | Harris et al. (2004b) | Harris et al. (2004b) | * | * | * | * |
|  | Tm61***** | Lithica, Menorca | Spain | Harris et al. (2004b) | Harris et al. (2004b) | Rato et al. (2010) | Rato et al. (2010) | * | * |
|  | Tm65 | Sierra Nevada | Spain | Harris et al. (2004a) | Harris et al. (2004a) | * | * | * | * |
|  | Tm127***** | Adra, Almería | Spain | Perera and Harris (2008) | Perera and Harris (2008) | Rato et al. (2010) | Rato et al. (2010) | * | * |
|  | Tm132 | Tamajón, Guadalajara | Spain | Perera and Harris (2008) | Perera and Harris (2008) | * | * | * | * |
|  | Tm133***** | Los Milanos, Huelva | Spain | Perera and Harris (2008) | Perera and Harris (2008) | * | * | * | * |
|  | Tm134***** | Matabichos, Huelva | Spain | Perera and Harris (2008) | Perera and Harris (2008) | * | * | * | * |
|  | Tm135***** | Matalascañas, Huelva | Spain | Perera and Harris (2008) | Perera and Harris (2008) | * | * | * | * |
|  | Tm136 | Fontanar, Jaén | Spain | Perera and Harris (2008) | Perera and Harris (2008) | * | * | * | * |
|  | Tm137***** | Lanjarón, Granada | Spain | Perera and Harris (2008) | Perera and Harris (2008) | * | * | * | * |
|  | Tm138 | Robledo, Albacete | Spain | Perera and Harris (2008) | Perera and Harris (2008) | * | * | * | * |
|  | Tm139 | Moratalla, Murcia | Spain | Perera and Harris (2008) | Perera and Harris (2008) | * | * | * | * |
|  | Tm141 | Berja/Castala, Almería | Spain | Perera and Harris (2008) | Perera and Harris (2008) | * | * | * | * |
|  | Tm142***** | Santa Ana, Jaén | Spain | Perera and Harris (2008) | Perera and Harris (2008) | * | * | * | * |
|  | Tm143 | Genave Station, Jaén | Spain | Perera and Harris (2008) | Perera and Harris (2008) | * | * | * | * |
|  | Tm144 | Collado, Albacete | Spain | Perera and Harris (2008) | Perera and Harris (2008) | * | * | * | * |
|  | Tm145 | Cíjara, Cáceres | Spain | Perera and Harris (2008) | Perera and Harris (2008) | * | * | * | * |
|  | Tm147 | Valdeinfierno, Córdoba | Spain | Perera and Harris (2008) | Perera and Harris (2008) | * | * | * | * |
|  | Tm149***** | Cerro del Hierro, Sevilla | Spain | Perera and Harris (2008) | Perera and Harris (2008) | * | * | * | * |
|  | Tm150***** | Palma del Río, Sevilla | Spain | Perera and Harris (2008) | Perera and Harris (2008) | * | * | * | * |
|  | Tm151***** | Medina Sidonia, Cádiz | Spain | Perera and Harris (2008) | Perera and Harris (2008) | * | * | * | * |
|  | Tm152 | Facinas, Cádiz | Spain | Perera and Harris (2008) | Perera and Harris (2008) | JQ301048 | JQ301153 | * | * |
|  | Tm153***** | La Sauceda, Málaga / Cádiz | Spain | Perera and Harris (2008) | Perera and Harris (2008) | * | * | * | * |
|  | Tm154***** | San Roque, Málaga / Cádiz | Spain | Perera and Harris (2008) | Perera and Harris (2008) | * | * | * | * |
|  | Tm294 | Galera, Granada | Spain | Perera and Harris (2008) | Perera and Harris (2008) | * | * | * | * |
|  | Tm300 | Amieiro-Valle do Tua, Amieiro | Portugal | Perera and Harris (2008) | Perera and Harris (2008) | * | * | * | * |
|  | Tm304***** | Chiclana de la Frontera, Cádiz | Spain | Perera and Harris (2008) | Perera and Harris (2008) | * | * | * | * |
|  | Tm308 | Benadalid, Málaga | Spain | Perera and Harris (2008) | Perera and Harris (2008) | * | * | * | * |
|  | Tm310 | Abreiro, Mirandela | Portugal | Perera and Harris (2008) | Perera and Harris (2008) | * | * | * | * |
|  | Tm311 | Picote, Miranda do Douro | Portugal | Perera and Harris (2008) | Perera and Harris (2008) | * | * | * | * |
|  | Tm328***** | Sta. Barbara, Amposta, Tarragona | Spain | Perera and Harris (2008) | Perera and Harris (2008) | * | * | * | * |
| *Tarentola mindiae* | SPM002968 | Qattara depression | Egypt | JQ300592 | JQ300790 | JQ301061 | JQ301144 | * | * |
| *Tarentola neglecta* | Ksar_1 | Ksar Ghilane | Tunisia | JQ300649 | JQ300998 | JQ301083 | JQ301152 | * | * |
|  | Ksar_2 | Ksar Ghilane | Tunisia | JQ300604 | JQ300853 | JQ301095 | JQ301182 | * | * |
|  | Ksar_3 | Ksar Ghilane | Tunisia | JQ300676 | JQ300837 | JQ301026 | JQ301196 | * | * |
|  | Ksar_4 | Ksar Ghilane | Tunisia | JQ300689 | JQ300978 | JQ301081 | JQ301160 | * | * |
|  | Ksar_5 | Ksar Ghilane | Tunisia | JQ300596 | JQ300874 | JQ301058 | JQ301159 | * | * |
|  | Ksar_6 | Ksar Ghilane | Tunisia | JQ300712 | JQ300801 | JQ301035 | JQ301181 | * | * |
| *Tarentola sp.* | BEV10121 | Massif d'Aïn Maïda, after Senalba | Algeria | JQ300621 | JQ300909 | * | * | * | * |
|  | DB303 | Conigli islet | Italy | Rato et al. (2010) | Rato et al. (2010) | Rato et al. (2010) | Rato et al. (2010) | * | * |
|  | DB304 | Conigli islet | Italy | Rato et al. (2010) | Rato et al. (2010) | * | * | * | * |
|  | DB3087 |  | Morocco | JQ300546 | JQ300899 | * | * | * | * |
|  | DB3088 |  | Morocco | JQ300738 | JQ300939 | * | * | * | * |
|  | DB3091 |  | Morocco | JQ300721 | JQ300924 | JQ301043 | JQ301157 | * | * |
|  | DB3098 |  | Morocco | JQ300622 | JQ300807 | * | * | * | * |
|  | DB3101 |  | Morocco | JQ300684 | JQ300915 | * | * | * | * |
|  | DB3126 |  | Morocco | JQ300755 | JQ300979 | * | * | * | * |
|  | DB3141 | Lampedusa | Italy | JQ300737 | JQ300811 | * | * | * | * |
|  | DB3158 | Conigli islet | Italy | JQ300634 | JQ300827 | JQ301037 | JQ301158 | * | * |
|  | DB3160 | Lampedusa | Italy | JQ300666 | JQ300870 | JQ301074 | JQ301120 | * | * |
|  | DB3181 | Conigli islet | Italy | JQ300571 | JQ300905 | * | * | * | * |
|  | DB3191 | NW point, Lampedusa | Italy | JQ300663 | JQ300855 | JQ301038 | JQ301111 | * | * |
|  | DB3194 | Cala Francese, Lampedusa | Italy | JQ300688 | JQ300840 | * | * | * | * |
|  | DB3195 | Cala Francese, Lampedusa | Italy | JQ300550 | JQ300882 | * | * | * | * |
|  | DB3196 | Cala Francese, Lampedusa | Italy | JQ300619 | JQ300823 | * | * | * | * |
|  | DB3198 | Cala Francese, Lampedusa | Italy | JQ300753 | JQ300872 | * | * | * | * |
|  | DB3199 | Cala Francese, Lampedusa | Italy | JQ300556 | JQ300841 | JQ301013 | JQ301172 | * | * |
|  | DB3201 | Lampedusa | Italy | JQ300759 | JQ300856 | * | * | * | * |
|  | DB3204 | Sanguedolce, Lampedusa | Italy | JQ300762 | JQ300776 | JQ301093 | JQ301205 | * | * |
|  | DB3208 | Sanguedolce, Lampedusa | Italy | JQ300700 | JQ300910 | JQ301021 | JQ301136 | * | * |
|  | DB3213 | Conigli islet | Italy | JQ300553 | JQ300847 | * | * | * | * |
|  | DB3214 | Conigli islet | Italy | JQ300734 | JQ300814 | * | * | * | * |
|  | DB3215 | Lampedusa | Italy | JQ300590 | JQ300995 | * | * | * | * |
|  | DB3264 |  | Morocco | JQ300745 | JQ300937 | * | * | * | * |
|  | DB3265 |  | Morocco | JQ300610 | JQ300943 | * | * | * | * |
|  | DB354 | Lampedusa | Italy | Rato et al. (2010) | Rato et al. (2010) | * | * | * | * |
|  | DB422 | Dougga | Tunisia | JQ300543 | JQ300916 | JQ301007 | JQ301188 | JQ301228 | JQ301340 |
|  | DB469 | Hassi Neguib, Djelfa | Algeria | JQ300691 | JQ300805 | * | * | * | * |
|  | DB470 | Theniet Djerrad, Bou Saada | Algeria | JQ300643 | JQ300969 | * | * | * | * |
|  | DB471 | Sougueur | Algeria | JQ300707 | JQ300949 | JQ301046 | JQ301113 | * | * |
|  | DB475 | Theniet el had National Park | Algeria | JQ300763 | JQ300956 | JQ301019 | JQ301114 | * | * |
|  | DB478 | Theniet Djerrad, Bou Saada | Algeria | JQ300574 | JQ300813 | * | * | * | * |
|  | DB483 | M'Doukal, Batna | Algeria | JQ300638 | JQ300772 | JQ301077 | JQ301151 | * | * |
|  | DB484 | M'Doukal, Batna | Algeria | JQ300701 | JQ300938 | JQ301032 | JQ301156 | * | * |
|  | DB489 | Belezma National Park, Batna | Algeria | JQ300568 | JQ300904 | JQ301102 | JQ301178 | * | * |
|  | DB9001 | Oued shili (31km from Tozeur) | Tunisia | JQ300682 | JQ300839 | JQ301020 | JQ301184 | * | * |
|  | DB9002 | Tozeur | Tunisia | JQ300648 | JQ300933 | * | * | * | * |
|  | DB9003 | Tozeur | Tunisia | JQ300644 | JQ300985 | * | * | * | * |
|  | DB9004 | Hammam (Close to Tozeur) | Tunisia | JQ300768 | JQ300984 | * | * | * | * |
|  | T1739 | Senalba, Djelfa | Algeria | JQ300683 | JQ300775 | * | * | * | * |
|  | T1740 | Senalba, Djelfa | Algeria | JQ300600 | JQ300964 | * | * | * | * |
|  | T1741 | Senalba, Djelfa | Algeria | JQ300711 | JQ300863 | * | * | * | * |
|  | T31-23 Taremaur1 | Chergui island (Kerkenah island) | Tunisia | JQ300613 | JQ300800 | * | * | * | * |
|  | T31-23 Taremaur2 | Chergui island (Kerkenah island) | Tunisia | JQ300697 | JQ300770 | * | * | * | * |
|  | T31-29 Taremaur1 | Sfax | Tunisia | JQ300687 | JQ300953 | * | * | * | * |
|  | T31-29 Taremaur2 | Sfax | Tunisia | JQ300724 | JQ300958 | JQ301054 | JQ301163 | * | * |
|  | T31-32 Taremaur1 | 140km from Sfax | Tunisia | JQ300575 | JQ300892 | JQ301012 | JQ301208 | * | * |
|  | T31-45 Taremaur1 | 13Km from Kasserine | Tunisia | JQ300576 | JQ300832 | * | * | * | * |
|  | Tm159 | Conigli islet | Italy | Harris et al. (2009) | Harris et al. (2009) | * | * | * | * |
|  | Tm213 | Lampedusa village | Italy | Harris et al. (2009) | Harris et al. (2009) | * | * | * | * |
|  | Tm216 | Conigli islet | Italy | Harris et al. (2009) | Harris et al. (2009) | * | * | * | * |
|  | Tm219 | Conigli islet | Italy | Harris et al. (2009) | Harris et al. (2009) | * | * | * | * |
|  | Tm220 | Conigli islet | Italy | Harris et al. (2009) | Harris et al. (2009) | * | * | * | * |
|  | Tm223 | Sanguedolce, Lampedusa | Italy | Harris et al. (2009) | Harris et al. (2009) | * | * | * | * |
|  | Tm224 | Sanguedolce, Lampedusa | Italy | Harris et al. (2009) | Harris et al. (2009) | * | * | * | * |
|  | Tm226 | Sanguedolce, Lampedusa | Italy | Harris et al. (2009) | Harris et al. (2009) | * | * | * | * |
